# Ultra-late relapse of acute promyelocytic leukemia 18 years after complete remission: a case report and literature review

**DOI:** 10.3389/fmed.2025.1651742

**Published:** 2025-09-15

**Authors:** Lu Gao, Yang An, Qian Li, Shuo Shi, Jie Zhou, Zuochen Du, Pei Huang, Mingqiang Ren, Yan Chen

**Affiliations:** ^1^Department of Hematology, Affiliated Hospital of Zunyi Medical University, Zunyi, China; ^2^Department of Pediatrics, Affiliated Hospital of Zunyi Medical University, Zunyi, China; ^3^Guizhou Children's Hospital, Zunyi, China; ^4^Department of Laboratory Medicine, Affiliated Hospital of Zunyi Medical University, Zunyi, China

**Keywords:** acute promyelocytic leukemia, arsenic trioxide, all-trans retinoic acid, ultra-late relapse, Case Report and Literature Review

## Abstract

Late relapse of acute promyelocytic leukemia (APL) is associated with high mortality rates. While APL typically shows a low incidence of relapse after achieving complete remission (CR) for more than 7 years, we report a rare case of APL relapse occurring 18 years after CR was achieved. The patient was successfully treated with a combination of arsenic trioxide (ATO) and all-trans retinoic acid (ATRA), leading to favorable outcomes. Additionally, we review our treatment experience and provide a comprehensive analysis of the existing literature, summarizing the characteristics of reported APL cases that relapsed after maintaining CR for over 7 years.

## Introduction

Acute promyelocytic leukemia (APL), characterized by the t(15,17)(q22;q12) translocation, is a hematologic emergency that is associated with high early mortality due to coagulopathy (1, 2). Modern therapies combining all-trans retinoic acid (ATRA) and arsenic trioxide (ATO) have led to complete remission (CR) rates exceeding 90% (3–6), with late relapses occurring more than 7 years post-CR being rare (7). This study presents an exceptional case of APL relapse occurring 18 years after the initial CR, successfully treated with ATO/ATRA-based therapy, and provides a comprehensive review of the current literature on ultra-late relapses in APL.

## Case presentation

### Initial presentation and treatment (1999)

In 1999, a 31-year-old woman was diagnosed with APL following evaluation for gingival bleeding and easy bruising. Laboratory studies at diagnosis revealed leukocytosis (white blood cell count, WBC: 13 × 10^9^/L), thrombocytopenia (platelet count: 20 × 10^9^/L), and bone marrow morphology showed a predominance of abnormal promyelocytes (80%). At that time, PML-RARA fusion testing was not yet part of routine clinical practice due to technological and logistical constraints, and thus molecular confirmation was not obtained. Induction therapy with daunorubicin (45 mg/m^2^/day for 3 days), cytarabine (100 mg/m^2^/day for 7 days), and ATRA (25 mg/m^2^/day for 28 days) led to complete hematologic remission. The patient subsequently received three cycles of consolidation chemotherapy (daunorubicin, cytarabine, and ATRA), followed by ATRA-based maintenance therapy (25 mg/m^2^/day for 14 days per month) over 3 years.

### Ultra-late relapse and reinduction (2017)

**Figure 1:**
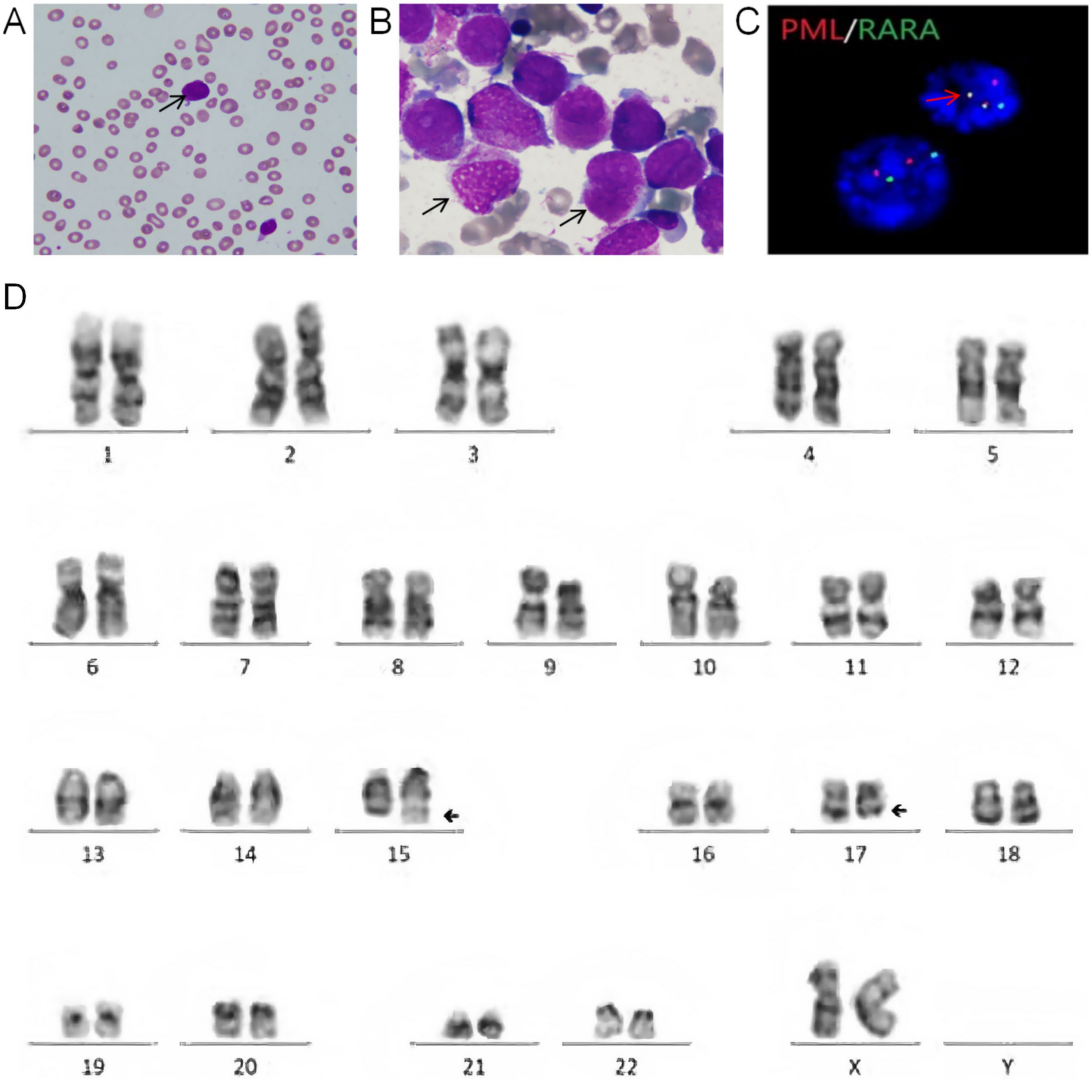
Diagnostic characteristics of late-relapse acute promyelocytic leukemia (APL). **(A)** Peripheral blood smear (Wright-Giemsa stain; 1,000 × magnification): Abnormal hypergranular promyelocytes. **(B)** Bone marrow aspirate (1,000 × magnification): Irregular promyelocytes exhibiting variable size, abundant cytoplasm, coarse azurophilic granules, and prominent Auer rods. **(C)** Fluorescence *in situ* hybridization (FISH): Positive PML-RARα fusion signals (yellow) using dual-color translocation probes. **(D)** Karyotype (G-banding): 46, XX, t(15,17)(q22;q12)—pathognomonic of APL per WHO classification.

Eighteen years later, the patient presented with acute cutaneous ecchymosis and hemorrhagic gingivitis. Laboratory findings revealed pancytopenia (WBC 0.9 × 10^9^/L with 30% blasts, platelets 41 × 10^9^/L) and marked hypofibrinogenemia (0.6 g/L). Bone marrow aspiration confirmed relapsed APL (Figures 1A,B), supported by immunophenotypic markers (CD33^+^, CD117^+^, cMPO^+^, HLA-DR^−^, CD34^−^), cytogenetic showing 46, XX, t(15,17)(q22;q12) [20 metaphases] (Figure 1D), and molecular evidence of PML-RARα fusion by FISH (Figure 1C). Reinduction therapy was initiated with ATRA (25 mg/m^2^/day) and ATO (0.16 mg/kg/day). On day 5, the patient developed differentiation syndrome characterized by hypoxemia, serosal effusions, and a 5-kg weight gain due to fluid overload, necessitating ATRA discontinuation and initiation of dexamethasone. By day 10, the patient developed disseminated intravascular coagulation (DIC), evidenced by severe thrombocytopenia (platelets < 20 × 10^9^/L, prolonged PT/APTT, fibrinogen <0.5 g/L, and FDP 111.7 μg/mL), requiring intensive supportive management. Despite these complications, dual -agent therapy was resumed and achieved morphologic remission (3% promyelocytes) by day 30.

### Consolidation and long-term outcomes

**Figure 2:**
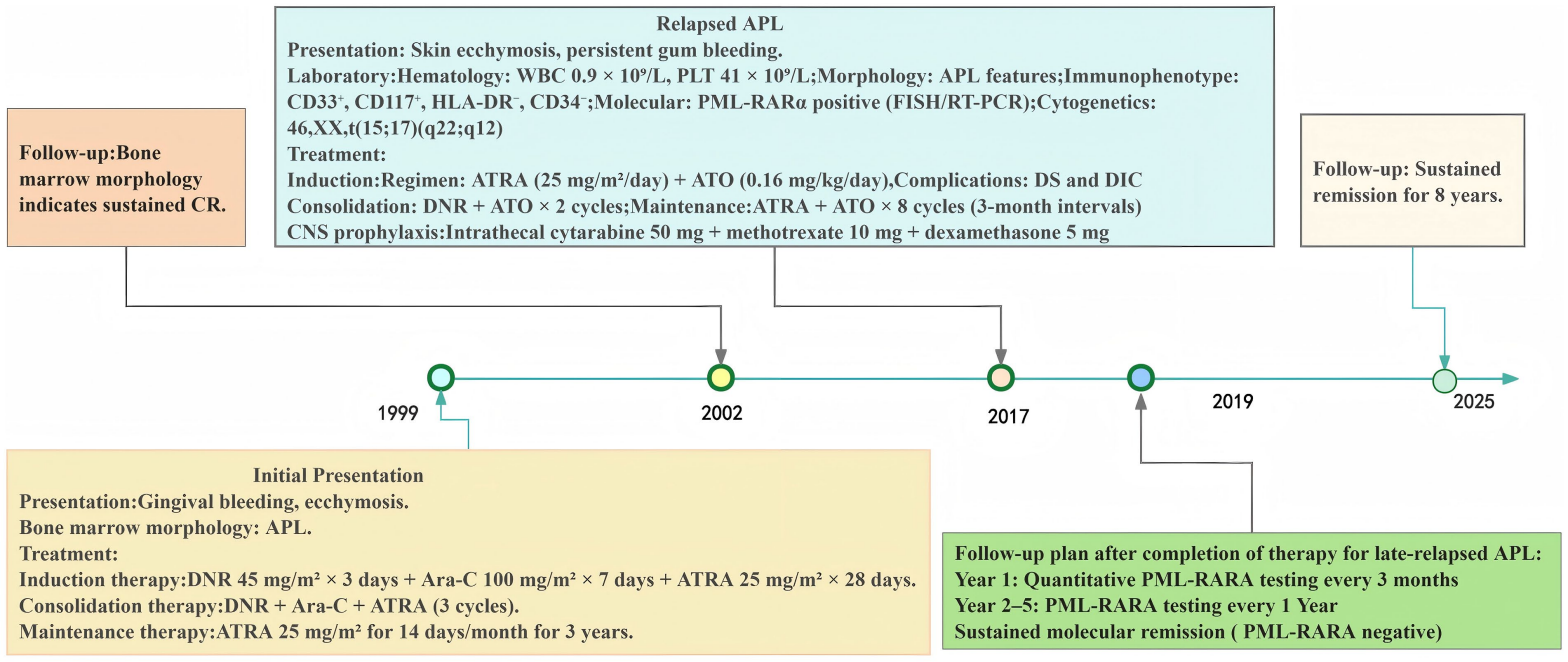
Diagnostic and therapeutic timeline for ultra-late relapsed APL. APL, acute promyelocytic leukemia; Ara-C, cytosine arabinoside; APTT, activated partial thromboplastin time; ATO, arsenic trioxide; ATRA, all-trans retinoic acid; CNSL, central nervous system leukemia; CR, complete remission; DS, differentiation syndrome; DIC, disseminated intravascular coagulation; DNR, daunorubicin; FDP, fibrin degradation products; FISH, fluorescence in situ hybridization; PLT, platelet; PT, prothrombin time; RT-PCR, reverse transcription polymerase chain reaction; WBC, white blood cell.

Two cycles of consolidation therapy with ATO and daunorubicin successfully eradicated detectable PML-RARα transcripts. Although the patient declined autologous hematopoietic stem cell transplantation, she completed a two-year maintenance regimen consisting of ATO and ATRA, administered in intermittent cycles as recommended for relapsed APL in published guidelines (8, 9), along with five prophylactic intrathecal administrations. Serial molecular monitoring during follow-up confirmed sustained molecular remission over a period of 8 years. This exceptional case underscores the potential for ultra-late APL relapse to remain curable with arsenic-based regimens, even 18 years of initial complete remission. A timeline summarizing the diagnostic and therapeutic course of this patient is presented in Figure 2.

### Literature review

We analyzed 11 reported cases of ultra-late APL relapse (defined as relapse ≥7 years after achieving complete remission), including the current case, to elucidate clinical and therapeutic characteristics (Table 1) (7, 10–16). The cohort consisted of 5 male and 6 female patients, with a median age at relapse of 34 years (range: 15–52 years). All patients had received induction and consolidation therapy incorporating ATRA at the time of initial diagnosis, in accordance with standard APL treatment principles. However, only two patients received ATRA-based maintenance therapy, while the remaining nine did not undergo maintenance treatment.

**Table 1:** Characteristics and outcomes of acute promyelocytic leukemia (APL) patients with ultra late relapse (>7 years post-remission).

Case no	Sex/age	Karyotype	PML/RARα type	Therapy at initial induction consolidation maintenance	Relapsed form	Time since diagnosis (years)	Immunophenotype relapse	PML/RARa type	Therapy at relapse induction consolidation maintenance	Follow-up (months)	Reference
1	F/43	t(15;17)(q22;q12)	Bcr-3	AIDA 2000 protocol ATRA+IDA 6MP+MTX+ATRA	Intra-parotid lymph nodes Molecular relapse	9	CD13+, CD33+, HLA-DR−	Bcr-3	ATO+ATRA+IDA/RT ATRA+ATO ATO+ATRA	Remission 12	Molica et al. (7)
2	F/42	t(15;17)(q22;q12)	PML-RARa FLT3-ITD	ATRA+DNR+Ara-C DNR+Ara-C	Intramedullary	17	NA	PML-RARa FLT3-D835	ATRA+DNR+Ara-C ATRA+ATO ATO+ATRA	Molecular remission 7	Zhang et al. (10)
3	F/52	t(15;17)(q22;q12-21)	PML-RARa	ATRA DNR+Ara-C	Intramedullary	11	NA	PML-RARa	ATRA+ATO DNR+Ara-C ATRA	Molecular remission 12	Zhan et al. (11)
4	M/30	t(15;17)(q22;q12)	Bcr1–2	AIDA protocol ATRA +IDA	Intramedullary	9.25	CD13+, CD33+, HLA-DR−	Bcr1–2	ATRA+IDA ATRA+IDA ATRA	Molecular remission 11	Ferrara et al. (12)
5	M/25	t(15;17)(q22;q12)	Bcr1–2	AIDA protocol ATRA	Intramedullary	7	CD13+, CD33+, HLA-DR−	Bcr1–2	ATRA+IDA ATRA+IDA ATRA	Molecular remission 32	Ferrara et al. (12)
6	M/24	t(15;17)(q22;q12)	Bcr1–3	ATRA +IDA ATRA +IDA ATRA	Mastoid cavity	15	CD13+, CD33+, CD34-, HLA-DR−	Bcr1–3	ATRA+ATO ATRA+ATO ATRA	Remission 72	Testi et al. (13)
7	M/52	t(15;17)(q22;q12)	PML-RARa	ATRA+DNR+Ara-C JALSG AML89 protocol	Intramedullary	17	CD13+, CD33+, CD38+, CD34-	PML-RARa	ATRA+DNR ATRA+ATO ATRA	Early death Legionella pneumonia 0.2	Sakurai et al. (14)
8	F/16	NA	Bcr1–3	LAP-0389	Intramedullary right mastoid	12.9	NA	NA	LAP-0389	Remission 2	Latagliata et al. (15)
9	F/30	NA	Bcr1–3	AIDA	Intramedullary	8.4	NA	NA	prot. 0191	Remission 29	Latagliata et al. (15)
10	M/15	t(15;17)(q22;q11-12)	PML-RARα	AML-BFM 98 protocol	Intramedullary	7	NA	PML-RARα	ATO ATO ATO	Molecular remission 48	Ebinger et al. (16)
11	F/49	t(15;17)(q22;q12)	NA	ATRA+DNR+Ara-C ATRA+DNR+Ara-C ATRA	Intramedullary	18	CD117+, CD33+, HLA-DR-, CD34-	PML-RARa	ATRA+ATO+DNR DNR+ATO ATRA+ATO	Molecular remission 96	Present case

Ara-C, cytarabine; ATO, arsenic trioxide; ATRA, all-trans retinoic acid; DNR, daunorubicin; F, female; IDA, idarubicin; M, male; 6MP, 6-mercaptopurine; MTX, methotrexate; NA, not available; RT, radiotherapy.

The mean interval from initial diagnosis to relapse was 12.3 years (range: 7–18 years), with the longest latency period being 18 years. All patients harbored the hallmark t(15,17)(q22;q12)/PML-RARα fusion gene characteristic of APL. Relapse site analysis revealed that eight patients experienced bone marrow relapse, while three presented with extramedullary relapse (one involving intraparotid lymph nodes, one in the mastoid cavity, and one in the right mastoid process). Immunophenotypic analysis, reported for six patients, demonstrated the classical APL profile: CD33^+^, CD117^+^, cMPO^+^, HLA-DR^−^, and CD34^−^. Notably, one patient exhibited an FLT3 mutation, transitioning from FLT3-ITD at initial diagnosis to FLT3-D835 at relapse, potentially contributing to leukemic persistence and clonal evolution.

In this limited cohort of ultra-late APL relapses, 10 out of 11 patients achieved remission following a variety of salvage regimens. Notably, all five patients who received arsenic-containing therapies (ATO ± ATRA) attained remission while other protocols, such as ATRA combined with chemotherapy demonstrated effectiveness. In 11 cases of late-relapsed APL, the duration of remission after achieving re-remission averaged 29.2 months (range: 0.2–96 months). Remarkably, the patient presented in our case report has remained in sustained remission for 8 years following reinduction therapy. To our knowledge, such long-term follow-up has not been previously documented in the context of ultra-late relapse, highlighting a significant gap in the existing literature regarding remission durability in this rare clinical scenario.

## Discussion

This case of APL relapse occurring 18 years after initial CR, represents one of the longest intervals reported to date and provides valuable insights into the phenomenon of ultra-late APL relapse. While the majority of relapses typically occur within 2 to 5 years of achieving CR (17), recurrence after such an extended latency period suggests the presence of unique biological mechanisms.

Relapse in APL remains a significant clinical challenge, particularly among patients presenting with high-risk features such as elevated WBC counts (18), FLT3 mutations (19, 20), and specific genetic alterations (21). FLT3 mutations are among the most common genetic alterations in APL, detected in up to 40% of cases (22). These mutations are frequently associated with leukocytosis and have been implicated in promoting leukemic infiltration into extramedullary sites, including the CNS (23). At the time of initial diagnosis, our patient had a WBC count exceeding 10 × 10^9^/L, consistent with hyperleukocytosis, which may have played a role in the eventual occurrence of ultra-late relapse.

The successful achievement of molecular remission through ATO and ATRA reinduction reaffirms this combination as the cornerstone of therapy for relapsed APL (24). Remarkably, despite an 18-year treatment-free interval, ATO and ATRA retained full therapeutic efficacy, achieving clearance of PML-RARα transcripts within two cycles of consolidation. The patient’s sustained remission over 8 years without undergoing hematopoietic stem cell transplantation further supports the role of ATO and ATRA as a definitive salvage strategy, particularly for patients who are ineligible for or decline transplantation.

The management of relapsed APL presents a complex clinical challenge, largely due to life-threatening complications such as differentiation syndrome (DS) (25) and disseminated intravascular coagulation (DIC) (26). These conditions are critical determinants of prognosis and require vigilant monitoring and prompt intervention during reinduction therapy. DS, which occurs in approximately 25% of APL patients treated with ATRA and ATO (25, 27), is characterized by systemic inflammation and cytokine dysregulation. This proinflammatory state can exacerbate the risk of DIC, a coagulopathy marked by simultaneous thrombosis and bleeding tendencies (28). In the present case, the patient developed rapid-onset hypoxemia and serositis, necessitating the immediate discontinuation of ATRA and the initiation of high-dose dexamethasone. Concurrently, refractory coagulopathy required aggressive fibrinogen replacement to manage severe DIC. The successful resolution of these complications highlights the importance of protocol-driven crisis management, including early cytokine suppression, goal-directed transfusion strategies, and maintenance of therapeutic intensity despite hematologic instability (29). The interplay between DS and DIC during reinduction underscores the need for proactive, multidisciplinary management strategies to mitigate complications and improve survival outcomes in relapsed APL.

Building upon our literature review, this analysis confirms that ultra-laterelapse of APL (≥7 years post-remission) remains exceptionally rare (7, 10–16). This rarity notwithstanding, the possibility of relapse beyond standard surveillance periods suggests that the duration of molecular monitoring in APL should be reconsidered. Our case represents one of the longest documented relapse intervals to date at 18 years, modestly exceeding the previously reported maximum of 17 years (10, 14). Although therapeutic approaches varied across the cohort, including ATO/ATRA-based salvage therapy (7, 10, 11), chemotherapy combined with ATRA (12), and hematopoietic stem cell transplantation (13), all regimens yielded favorable outcomes, with 10 out of 11 patients achieving remission. Notably, our patient, treated with ATO/ATRA, remains in remission 8 years post-salvage therapy, underscoring the potential for durable responses even in the context of extreme relapse latency. Currently, the longest reported post-relapse follow-up duration in the literature is 6 years (13). Our case offers the longest systematically documented remission duration following an ultra-late relapse. This finding supports with existing evidence that late-relapse APL retains sensitivity to conventional salvage regimens, including arsenic-based therapy (24). However, the heterogeneity of treatments and the absence of standardized consolidation and maintenance strategies underscore the pressing need for further studies to establish structured management protocols for this distinct subset of patients.

## Conclusion

This study highlights three key clinical implications. First, the duration of molecular surveillance in APL should be reconsidered, as the risk of ultra-late relapse may exceed beyond current monitoring timeframes. Second, ATO/ATRA-based regimens may be reasonably initiated empirically in cases of suspected relapse, given their efficacy in reported cases, though further validation is warranted. Third, ultra-late relapsed APL represents a distinct clinical entity, emphasizing the need for further investigation to optimize treatment strategies and define long-term management approaches. Together, these findings call for heightened clinical awareness and collaborative efforts to address the unmet needs of this rare but consequential patient subset.

## Data Availability

The original contributions presented in the study are included in the article/supplementary material, further inquiries can be directed to the corresponding authors.
